# The Inclusion Principles of Human Embryos in the WOW-Based Time-Lapse System: A Retrospective Cohort Study

**DOI:** 10.3389/fendo.2021.549216

**Published:** 2021-07-26

**Authors:** Yuqiong Wang, Sheng Wang, Xilin Qian, Yanrong Kuai, Yang Xu

**Affiliations:** The Department of Assisted Reproduction, Peking University First Hospital, Beijing, China

**Keywords:** time-lapse system (TLS), well-of-the-well (WOW), embryo group culture, embryo secretome, blastocyst formation

## Abstract

A time-lapse system (TLS) with a well-of-the-well (WOW) dish, which allows individual identification and the possibility of autocrine and paracrine signaling between group-cultured embryos, has been widely used in clinic. However, there is a need to re-think the inclusion principles of human embryos in WOW-based TLS, especially for grade IV (G4) embryos, which are considered to potentially have detrimental effects on surrounding embryos. Here, we carried out a single-center, large-cohort, retrospective study, comprising 303 patients undergoing IVF (148 cases) and ICSI (155 cases), with a total of 3282 embryos, to compare embryonic development until the blastocyst stage in the group culture system with or without G4 embryos. Further, LC-MS/MS was used to analyze the G1-G4 embryo secretome to understand the influence of G4 embryos on the group culture microenvironment. We proved that polypronuclear (PPN) embryos positively contribute to the development of the neighboring embryos through secretion of ILIAP, ITI-H4, and keratin. Existence of more than one G4 embryo had a negative effect on the other embryos (p < 0.05). Moreover, G4 embryos were found to secrete KLKB1 and VTDB, which might harm the neighboring embryos. Thus, our study clarified that when embryos are subjected to group culture in WOW-based TLS, the PPN-derived embryos need not be removed, and it is important to ensure that no more than one G4 embryo is present to avoid negative effects on the neighboring embryos.

## Introduction

The time-lapse system (TLS) is an effective method for continuous imaging of embryonic development *in vitro*; it not only provides detailed information of the developmental kinetics and cleavage patterns throughout the culture period (1, 2), but also maintains a stable and optimal embryo culture environment without having to remove the culture dish from the incubator (1, 3). At the same time, it greatly liberates embryologists from cumbersome clinical procedures, and is becoming increasingly popular (3).

The commonly used embryo culture dish for TLS is the so-called well-of-the-well (WOW) dish. This type of culture dish uses narrow and deep microwells to designate the place of each embryo within a close distance from one another to facilitate autocrine signaling and enable paracrine communication between sibling embryos, thus combining the beneficial effects of individual and group culture (4, 5). It was the first system to realize individual embryo identification and the possibility of autocrine and paracrine-mediated signaling between group-cultured embryos (4, 6–8). It is hypothesized that one benefit of group embryo culture is that it allows embryos to modify their microenvironment in an autocrine manner to exert an effect on themselves and/or on neighboring embryos (9), which may provide mutual ‘‘corrective’’ factors that compensate for insufficiency or imbalance in the individual embryo. These factors may act as growth factors or survival factors, protecting the embryos from subsequent cell death (10). Potential embryo-derived secretory factors include members of the insulin and insulin-like growth factor and tumor necrosis factor families, epidermal growth factors, fibroblast growth factors, and platelet-derived growth factors (11, 12).

However, some factors derived from poor-quality embryos may have negative influences on the development of surrounding embryos. It was reported that group culture of high-quality human embryos, rather than randomly grouped embryos regardless of embryo quality, may benefit blastocyst formation, owing to the secretion of beneficial factors by good embryos, or removal of detrimental factors from poor embryos (9). Unfortunately, we lack evidence regarding what the detrimental factors from poor embryos are and further analysis is necessary to determine the effect of these proteins on aspects such as development of neighboring embryos, clinical outcome, and even the health of the offspring (13).

During traditional group embryo culture, grade IV (G4) embryos are abandoned because they usually consist of polypronuclear (PPN) embryos, embryos with > 50% fragmentation, and development-arrested embryos (14); even if these G4 embryos develop into blastocysts, they are thought to be unsuitable for transplantation. However, in the era of TLS, that allows individual embryo identification in a WOW group culture system, we need to re-think the rule for inclusion of embryos, whether it is necessary to remove the G4 embryos specifically and determine whether G4 embryos secrete detrimental factors that harm the microenvironment of the group culture.

Hence, we conducted a single-center, large-cohort, retrospective study to compare embryonic development in the group culture system with or without G4 embryos to clarify the inclusion principle of human embryos in the WOW-based TLS system. Based on the findings from Stokes et al. that the benefit of group culture is usually visible after prolonged culture of human embryos to the blastocyst stage, which might be because group culture confers a greater advantage on development post genome activation (10), the quality of blastocysts from the two groups was mainly compared and used as an important index to evaluate the microenvironment of the group culture in the TLS.

## Methods

### Study Design

This study was a single-center, large-cohort, retrospective study. Blastocyst formation rate and blastocyst quality were used to evaluate the embryo microenvironment. Liquid chromatography-tandem mass spectrometry (LC-MS/MS) was used to analyze the G1–G4 embryo secretome to understand the influence of G4 embryos on the group culture microenvironment.

### Study Population

We included 303 patients undergoing *in-vitro* fertilization (IVF) (148 cases) and intracytoplasmic sperm injection (ICSI) (155 cases), involving 3282 embryos in total, from the Department of Reproductive Center, Perking University First hospital, Beijing city, China from September 1, 2018 to December 30, 2019.

The patients were referred to our clinic with a history of more than 1 year of infertility. Patients had the following primary etiologies for their infertility: male factor, tubal factor, unexplained, endometriosis, anovulation, and polycystic ovary syndrome.

#### Patients Selection

Inclusion criteria: Age 25~40 years old, and the number of retrieved oocytes 8-20. Exclusion criteria: Presence of organic ovarian diseases, such as ovarian cysts, ovarian benign tumors; oral contraceptives, metformin and other drugs that affect hormone levels and metabolism-related drugs within the past three months; age > 40 years old; the number of retrieved oocytes was less than 8 or more than 20.

Ovarian stimulation was performed with gonadotropin-releasing hormone (GnRH) analog downregulation utilizing our long or microflare protocols with recombinant follicle-stimulating hormone and luteinizing hormone or human menopausal gonadotrophin, dose adjusted according to body mass index. During the ovarian stimulation regimen, the patients underwent transvaginal ultrasonographic evaluation of endometrial thickness and measurement of follicular number and size. Ovulation was induced with recombinant human chorionic gonadotropin (hCG) when three or more follicles were at least 18 mm in their greatest diameter.

### Oocyte Retrieval and *In-Vitro* Fertilization

The oocyte retrievals were done by transvaginal aspiration under ultrasound guidance after 36 h from the hCG administration. The follicles were flushed by G-MOPS (Vitrolife) medium. After retrieval, oocytes were rapidly isolated from follicular fluid, and cultured in G-IVF medium (Vitrolife). Oocytes were inseminated 3–5 h later by classical IVF (mean concentration of 200 000 motile spermatozoa/mL) or by ICSI. Spermatozoa for IVF and ICSI were prepared with the swim-up technique and density gradient centrifugation method, respectively. For ICSI, cumulus cells needed to be removed by hyaluronidase (Vitrolife), and then the single motile spermatozoan with the best morphologic appearance was injected into each mature oocyte.

### Embryo Culture

**Figure 1:**
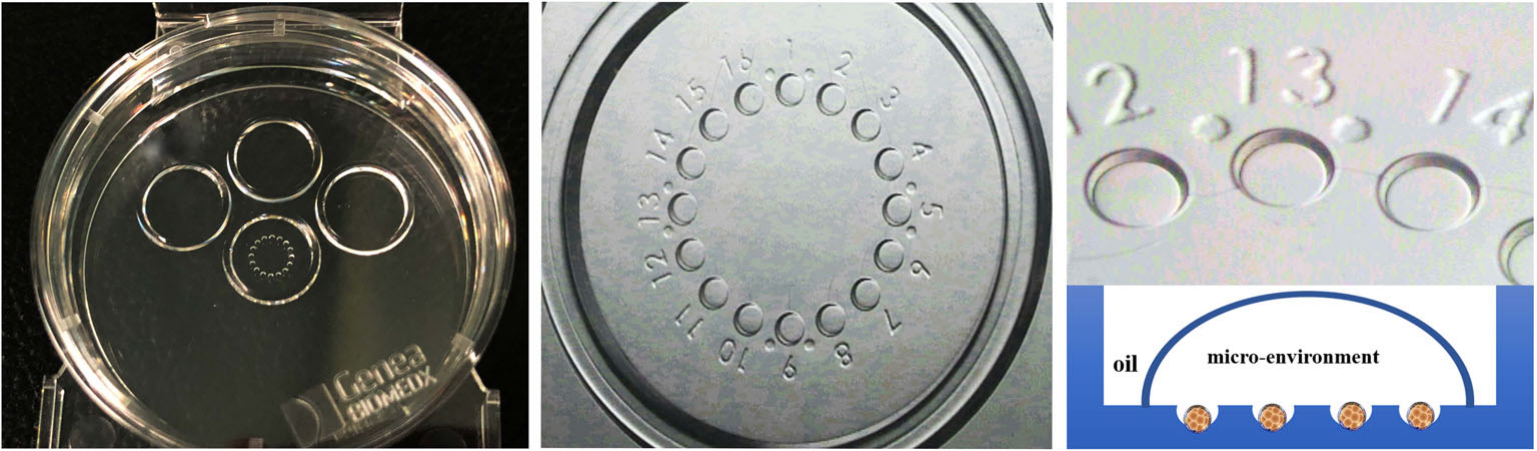
The WOW-based 16-microwell GERI dish. The WOW-based dish uses narrow, deep microwells to designate the place of each embryo within a close distance from each other, to facilitate autocrine signaling and enable paracrine communication between sibling embryos.

After IVF or ICSI, the oocytes and/or zygotes were seeded in groups using 16-microwell GERI dishes (GENEA BIOMEDX) (**Figure 1**) in a small droplet of preincubated G-1 Plus (Vitrolife) culture medium (80 μl) covered by mineral oil (3 ml) (Ovoil, Vitrolife). The microwell plate was placed on the time-lapse system (GERI) and fertilized oocytes were scored for pronuclei on day 1 (usually 18-19 h post-insemination).

### Day 3 Embryo Classification

Embryos were classified on day 3 into four categories as follows: Grade I (G1): Embryos (7–9 cells) with even, regular, spherical blastomeres with no or < 5% fragmentation; grade II (G2): Embryos (7–9 cells) with regular, spherical blastomeres, with < 20% fragmentation; grade III (G3): Embryos (≥ 4 cells) with uneven shaped blastomeres, with < 50% fragmentation; and grade IV (G4): Embryos (< 4 cells) with unequal, dark blastomeres (develop arrest) or despite more than four cells with unequal, dark blastomeres, a fragmentation of > 50%. Next, the embryos derived from PPN-fertilized eggs (5) were directly marked as G4 regardless of their cells or fragmentation.

The embryos were graded by SW and YRK, independently. And to avoid subjective factors, YQW only conducted the data analysis and did not take part in the work of embryo grading.

### Blastocyst Classification

On days 5-6, blastocyst formation was determined and each blastocyst was graded using the system of Gardner and Schoolcraft. Briefly, blastocysts were given an alphanumeric score from 1 to 6, based on their degree of expansion and hatching status, as follows: 1, early blastocyst, the blastocoel being less than half the volume of the embryo; 2, blastocyst, the blastocoel being half or greater than half of the volume of the embryo; 3, full blastocyst, the blastocoel completely fills the embryos; 4, expanded blastocyst, the blastocoel volume is larger than that of the early embryos and the zona is thinner than before; 5, hatching blastocyst, the trophectoderm has started to herniate through the zona; and 6, hatched blastocyst, the blastocyst has completely escaped from the zona.

The development of the inner cell mass (ICM) was graded as follows: A, tightly packed, many cells; B, loosely grouped, several cells; or C, very few cells. The trophectoderm was assessed as follows: A, many cells forming a cohesive epithelium; B, few cells forming a loose epithelium; or C, very few large cells. The high-quality blastocysts were those with a score ≥ 3BB, and the criterion for cryopreservation of blastocysts was that blastocysts had a score of ≥ 3BC at days 5 or 6.

### LC-MS/MS Analysis

The culture medium first underwent enzymolysis, and was then separated by capillary high performance liquid chromatography and analyzed by a Q-Exactive plus mass spectrometer (Thermo Scientific). The conditions were: analysis duration, 60 min; detection mode, calibrated by standard calibration solution before use; scanning range of precursor ion, 300-1800 m/z; mass spectrometry scanning mode, data-dependent acquisition mode (DDA); the strongest 10 fragment spectrogram (MS2 scan) were collected after each full scan; fragmentation mode, high energy collision dissociation (HCD); NEC energy, 27; and dynamic exclusion time, 40 s. The resolution was 70000 when the m/z of MS1 was 200, AGC target was set to 3e6, and the maximum injection time was 50 ms; MS2 resolution was set to 17500, AGC target was set to 5e5, and the maximum injection time was 50 ms. We have shared our data on the iProX platform (https://iprox.org/): project ID IPX0002563000, PXD022232.

### Gene Ontology (GO) Enrichment Analysis

To give an insight into the functions of the secreted protein, we performed GO enrichment analysis, and the significantly changed proteins from G4 and PPN embryo culture medium compared with the G1-G3 embryos were analyzed separately by GSEA.

### Statistical Analysis

All the analyses were performed with the statistical software packages of R (http://www.R-project.org, The R Foundation) and EmpowerStats (http://www.empowerstats.com, X&Y Solutions, Inc, Boston, MA). The continuous variables in our study, such as blastocyst formation rate, are presented as means and standard deviation (SD). Student’s t test or one-way analysis of variance was used to identify significant differences in quantitative variables. Categorical data are shown presented as percentage (%). Chi-square tests were used to identify significant differences in categorical data. Multivariate regression analysis was used to explore the influence of G4 embryos on the development of other embryos in the group culture system. Three models were conducted: model 1, no covariates were adjusted; model 2, only adjusted for female’s age (y), basal serum AMH (mIU/ml), BMI (kg/cm3), no. of retrieved oocytes, fertilization rate; model 3, all covariates presented in **Table 1** were adjusted. To analyze the effect of G4 embryos on the co-culture system of IVF or ICSI, subgroup analyses were performed using stratified linear regression models. As the number of embryos in one GERI dish would influence the blastocyst formation rate, we conducted subgroup analysis on embryo densities co-cultured in the same GERI dish. We divided that continuous variable into three groups, and calculated the P for trend, which presented the difference in the impact of G4 embryos on the co-culture environment under different embryo densities. A two-tailed P value <0.05 was considered statistically significant.

**Table 1:** Baseline characteristics of selected participants.

Characteristics of the study groups	Without G4 embryos (A)	With G4 embryos		P-value
		With polypronuclear embryos (B)	Without polypronuclear embryos (C)	
**No. of patients (n)**	131	77	95	
**Female’s age (y)**	31.11 ± 3.86	31.86 ± 3.85	31.87 ± 4.43	0.278
**Basal serum AMH (mIU/ml)**	4.05 ± 2.88	4.14 ± 3.00	4.06 ± 2.71	0.975
**BMI (kg/cm^3^)**	22.47 ± 3.88	21.59 ± 6.40	21.98 ± 4.78	0.442
**No. of retrieved oocytes (n)**	16.95 ± 7.40	18.77 ± 7.26	17.24 ± 7.11	0.210
**Fertilization rate**	0.67 ± 0.20	0.83 ± 0.18	0.69 ± 0.19	<0.001
**IVF OR ICSI**				<0.001
**IVF**	53 (40.46%)	72 (93.51%)	23 (24.21%)	
**ICSI**	78 (59.54%)	5 (6.49%)	72 (75.79%)	
**Ovulation inducing scheme**				0.629
**GnRH antagonist**	70 (53.44%)	42 (54.55%)	47 (49.47%)	
**Long luteal GnRHa**	60 (45.80%)	35 (45.45%)	47 (49.47%)	
**Others**	1 (0.76%)	0 (0.00%)	1 (1.05%)	

## Results

### Baseline Characteristics of Selected Participants

A total of 303 participants (3282 embryos) were selected for the final data analysis. The baseline characteristics of these selected participants are shown in **Table 1**. No statistically significant differences were detected in age, body mass index, number of retrieved oocytes, and ovulation inducing scheme (all p values > 0.05). However, the group with PPN embryos showed a higher fertilization rate (p < 0.05), because PPN embryos are usually present in the IVF cycle, whose fertilization rate is higher than that of ICSI.

### PPN Embryos Contribute to Improving the Development of the Neighboring Embryos in WOW-Based TLS

To avoid interference due to the above-mentioned reason, we conducted subgroup analysis (IVF or ICSI) to explore the influence of PPN embryos on the development of other embryos in the group culture system. The effect sizes (β) and 95% confidence intervals are listed in **Table 2**. In the IVF cycles, the group with PPN embryos showed a higher blastocyst formation rate, transplantable blastocyst formation rate, high-quality blastocyst formation rate, and effect sizes; for example, the effect size of 0.15 for blastocyst formation rate indicated that the difference in every unit of PPN embryos presented in the group culture system was associated with a 15% increase in blastocyst formation rate [0.15, 95%CI (0.02, 0.27)]. In other words, the PPN embryos present in the group-cultured microwell system contributed to the development of other embryos. However, in the ICSI cycles, there were no significant differences based on presence or absence of PPN embryos; this may be due to the following reasons: the occurrence probability of PPN embryos in the ICSI cycles is very low (only 5 cases in this study), due to which the statistics are not reliable. Furthermore, the mechanism of PPN formation in IVF and ICSI cycles are different (15): PPN zygote formation in IVF mostly depends on polyspermia. As a single sperm is injected into the oocyte during ICSI, the reason for PPN formation in ICSI is unclear; one of the explanation might be the inability to extrude the second polar body (2PB) (16). So the conclusion applies narrowly to IVF cycles.

**Table 2:** Subgroup analysis to explore the influence of PPN embryos on the development of other embryos in the group culture system.

Variable	IVF		ICSI	
	Group A	Group B	Group A	Group B
		effect size (95% CI)		effect size (95% CI)
		p value		p value
**Number**	53	72	78	5
**Blastocyst formation rate**	Ref	0.15 (0.02, 0.27) 0.0212	Ref	0.20 (-0.08, 0.47) 0.1669
**Transplantable blastocyst formation rate**	Ref	0.17 (0.05, 0.29) 0.0082	Ref	0.01 (-0.07, 0.28) 0.9551
**High quality blastocyst formation rate**	Ref	0.14 (0.03, 0.26) 0.0159	Ref	0.03 (-0.12, 0.19) 0.6832

We constructed three models to analyze the independent effects of PPN embryos on the development of other embryos in the group culture system in IVF cycles: unadjusted model (model 1), minimum-adjusted model (model 2), and fully-adjusted model (model 3) (adjusted for all covariates presented in **Table 1**). As shown in **Table 3**, for each Y value (blastocyst formation rate, transplantable blastocyst formation rate, high-quality blastocyst formation rate), the effect sizes (β) derived from the three models were similar and had a p < 0.05, which partly prove the stability of the above conclusion. Together, these results indicate that when the embryos go through group culture in the WOW-based TLS, the PPN embryos need not be abandoned, as the embryos co-cultured with the PPN embryos may benefit from it.

**Table 3:** Multivariate regression analysis to explore the influence of PPN embryos on the development of other embryos in the group culture system.

	Group A	Group B		
		Model 1	Model 2	Model 3
**Blastocyst formation rate**	Ref	0.15 (0.02, 0.27) 0.0212	0.15 (0.02, 0.28) 0.0256	0.15 (0.02, 0.28) 0.0253
**Transplantable blastocyst formation rate**	Ref	0.17 (0.05, 0.29) 0.0082	0.16 (0.04, 0.29) 0.0104	0.19 (0.06, 0.31) 0.0036
**High quality blastocyst formation rate**	Ref	0.14 (0.03, 0.26) 0.0159	0.14 (0.03, 0.26) 0.0175	0.15 (0.04, 0.27) 0.0109

The data in the table: effect size (95% CI) p value.

Unadjusted model (model 1), minimum-adjusted model (model 2), fully-adjusted model (model 3).

Adjusted factors: Female’s age (y); basal serum AMH (mIU/ml); BMI (kg/cm3); no. of retrieved oocytes; fertilization rate.

Group A: control group, means that there are no grade IV embryos; group B means that there are polypronuclear embryos in the WOW-based TLS system.

### G4 Embryos Might Have a Negative Influence on the Development of the Neighboring Embryos in WOW-Based TLS

In addition to PPN embryos, the G4 embryos also consist of embryos with fragmentation exceeding 50% and development-arrested embryos. Therefore, we next explored the effect of these “other G4 embryos” in the same manner mentioned above for the PPN embryos. As shown in **Table 4**, the results in IVF and ICSI cycles were similar. When there were no more than (≤) one G4 embryo (except PPN-derived), it had no effect on other embryos (p > 0.05). However, the existence of more than one G4 embryo in the group culture system negatively affected the other embryos (p < 0.05).

**Table 4:** Subgroup analysis to explore the influence of grade IV embryos on the development of other embryos in the group culture system.

	Groups	No.	Blastocyst formation rate	Transplantable blastocyst formation rate	High quality blastocyst formation rate
**IVF**	A	53	Ref	Ref	Ref
	C1	11	-0.03 (-0.24, 0.17) 0.7473	0.02 (-0.18, 0.22) 0.8259	0.01 (-0.18, 0.20) 0.9108
	C2	12	-0.26 (-0.46, -0.07) 0.0099	-0.26 (-0.45, -0.06) 0.0106	-0.18 (-0.36, 0.01) 0.0424
**ICSI**	A	78	Ref	Ref	Ref
	C1	50	0.06 (-0.05, 0.17) 0.2785	0.05 (-0.04, 0.15) 0.2811	0.01 (-0.05, 0.07) 0.7781
	C2	22	-0.07 (-0.22, 0.07) 0.0322	-0.09 (-0.22, 0.04) 0.0169	-0.03 (-0.11, 0.06) 0.05177

The data in the table: effect size (95% CI) p value.

The number of embryos co-cultured in one GERI dish could influence the blastocyst formation rate (5). To reduce the interference, we conducted subgroup analysis. As shown in the **Supplementary Table 1**, we divided the number of embryos in the GERI dishes into three groups, we found that the trend of the three subgroups were basically the same: more than one G4 embryo had a negative effect on the other embryos (p < 0.05).

And we further used the three models to analyze the independent effects of the G4 embryos on the development of other embryos in the group culture system. The results, shown in **Table 5**, also confirmed the negative effect. Thus, during group culture in WOW-based TLS, the number of G4 embryos (except PPN-derived) should be no more than (≤) 1, otherwise it might harm the development of other embryos.

**Table 5:** Multivariate regression analysis to explore the influence of grade IV embryos on the development of other embryos in the group culture system.

	**Model 1**	**Model 2**	**Model 3**
**Y= Blastocyst formation rate**			
**Groups**			
**A**	Ref	Ref	Ref
**C1**	0.02 (-0.07, 0.11) 0.6844	0.04 (-0.05, 0.13) 0.3894	0.04 (-0.05, 0.13) 0.3764
**C2**	-0.15 (-0.26, -0.03) 0.0161	-0.18 (-0.30, -0.06) 0.0025	-0.18 (-0.30, -0.07) 0.0018
**Y= Transplantable blastocyst formation rate**			
**Groups**			
**A**	Ref	Ref	Ref
**C1**	0.03 (-0.06, 0.12) 0.5716	0.05 (-0.04, 0.14) 0.2813	0.04 (-0.04, 0.13) 0.3159
**C2**	-0.15 (-0.27, -0.04) 0.0067	-0.19 (-0.30, -0.08) 0.0007	-0.20 (-0.30, -0.09) 0.0005
**Y= High quality blastocyst formation rate**			
**Groups**			
**A**	Ref	Ref	Ref
**C1**	-0.02 (-0.10, 0.05) 0.5682	0.01 (-0.06, 0.08) 0.8081	0.00 (-0.07, 0.07) 0.9410
**C2**	-0.09 (-0.18, 0.01) 0.0665	-0.12 (-0.21, -0.03) 0.0086	-0.12 (-0.21, -0.03) 0.0082

The data in the table: effect size (95% CI) p value.

Unadjusted model (model 1), minimum-adjusted model (model 2), fully-adjusted model (model 3).

Adjusted factors:Female’s age (y); basal serum AMH (mIU/ml); BMI (kg/cm3); no. of retrieved oocytes; fertilization rate.

Group A: control group, means that there are no grade IV embryos; group C1 means that there are fewer than one grade IV embryo (except polypronuclear embryos); group C2 means that there are more than one grade IV embryo (except polypronuclear embryo) in the WOW-based TLS system.

### The Secretome of G4 Embryos

**Figure 2:**
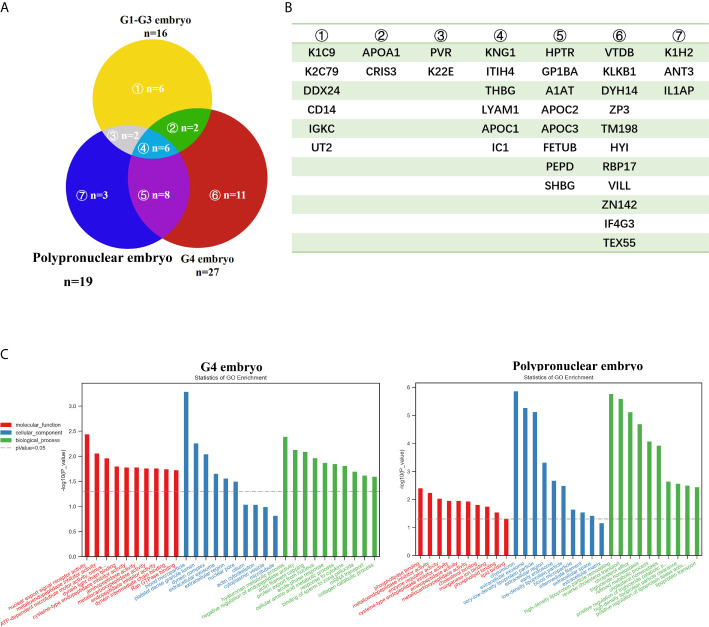
The secretome analysis of embryos with different grades. **(A, B)** Venn graph of the secretome of embryos with different grades, and the corresponding protein names of each part. **(C)** GO analysis of the secretome of embryos with different grades.

To determine the secretory proteins produced by the embryos and their potential influence on embryo development, we collected the culture medium in the micro-drops, which contained a single embryo. The G1-G2 embryo-, G3 embryo-, G4 embryo (except the PPN)-, and the PPN embryo-derived media were collected separately. These were then subjected to LC-MS/MS analysis to determine the secretome of the different quality embryos. As shown in **Figure 2A**, the G4 embryo-derived medium consisted of 27 proteins in all and 11 proteins that were unique to the G4 embryo (**Figure 2B**), which was more complex than the other groups. Moreover, GO analysis indicated that the G4 embryo-secreted proteins were related to acute phase response (**Figure 2C**), which might explain the negative influence of these embryos on the neighboring embryos. Notably, the PPN embryo-secreted proteins were related to lipid metabolism and synthesis (**Figure 2C**). And this might explain their contribution toward improving the development of their neighboring embryos in the WOW-based TLS. And the extracellular exosomes components were found both in the G4 and PPN embryos which suggest one possible idea: the secreted proteins may be encapsulated in exosomes, and exert their effect on the co-cultured embryos. However deep research is warranted to support this hypothesis.

## Discussion

The WOW-based TLS system has greatly improved the environment for embryo culture *in vitro* and facilitates clinical operation. Importantly, it allows group culture during the whole process from zygotes to blastocysts, and especially allows G4 embryos to share the same microenvironment with other embryos. Considering the increased widespread use of this system, there is a need to establish the inclusion principle for embryos under this system to create an optimal microenvironment for embryo culture *in vitro*.

Based on our single-center cohort study, we firstly identified that there is no need for PPN embryos to be excluded when the embryos are subjected to group culture in the WOW-based TLS system. In fact, it might be desirable to include them because the embryos co-cultured with PPN embryos may benefit from it. Secondly, our analysis suggested that the number of G4 embryos should be no more than (≤) one, as higher numbers can exert a negative effect on other neighboring embryos.

We further used the LC-MS/MS technique to identify the potential autocrine/paracrine factors released by embryos that could be responsible for the observed results. Although the actual process of secretory protein production by the different embryos and their subsequent function remain unclear, our analysis revealed some proteins that may have potential influence on the development of embryos. As shown in **Supplementary Table 2**, the inter-alpha-trypsin inhibitor heavy chain H4 (ITI-H4) was commonly shared by embryos of all grades, and might play positive roles in the maintenance of Th1/Th2 balance, contributing to anti-inflammation processes (17). In contrast, the plasma kallikrein (KLKB1) was detected only in the G4 embryo-derived medium. KLKB1 was reported to promote the transformation of the ITI-H4 longer form to its shorter form ITI-H4 (ΔN688), which is a controversial inflammatory factor that has been associated with recurrent pregnancy loss (17). The second possible detrimental factor identified in the G4 embryo-derived medium was vitamin D-binding protein (VTDB), which was reported to have negative correlations with fertility ranking in bull plasm (18). If these G4 embryo-derived proteins are taken up by the embryos in the microenvironment, they could have a wide range of effects on embryo development. Hence, it would be better to ensure that there is no more than one G4 embryo in the WOW-based TLS.

As for the PPN embryos, we found the interleukin-1 receptor accessory protein (IL1AP) to be specifically secreted by the PPN embryos. There are two forms of ILIAP: transmembrane form and soluble form. The soluble form of ILIAP is reported to be an inhibitor of IL-1 by directly interacting with IL-1RI to abolish its capacity to transduce a signal. Thus, together with the ITI-H4, the ILIAP secreted by the PPN embryos would inhibit inflammation, which might contribute to the better development of embryos in the same group culture system (19). Moreover, the PPN embryos and G1-G3 embryos were all found to secrete keratin proteins (KRT2, KRT32, KRT79, KRT9), which form a complex dynamic network that controls cell architecture, cell adhesion, cell migration, and cell differentiation (20, 21). Keratin-deficient mouse embryos were reported to die from severe growth retardation or restricted cytolysis in the trophoblast layer (21, 22), and keratins showed a significantly low presence in the low-hatched chicken embryo group, indicating that keratin deficiency could be closely related to early chicken embryo development.

**Figure 3:**
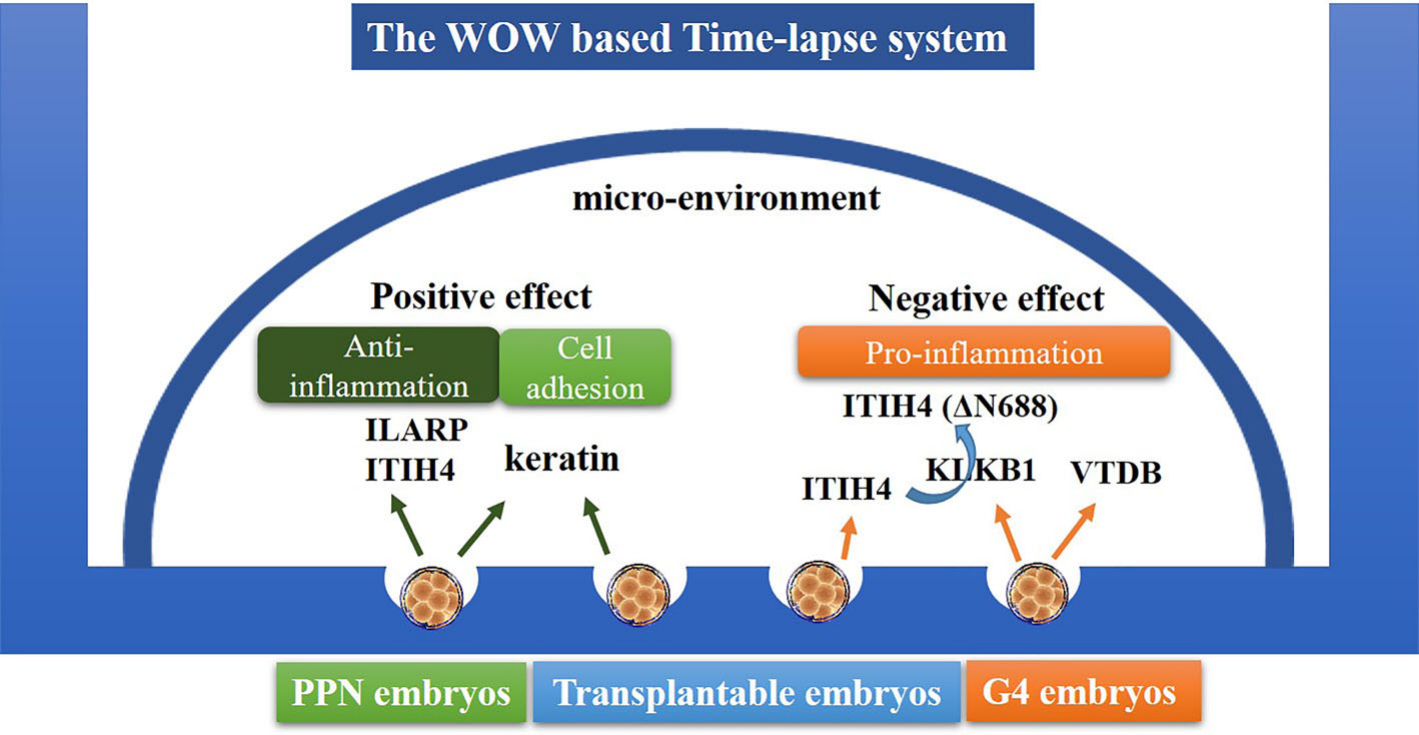
Schematic diagram of the interaction between embryos through autocrine/paracrine proteins. In the WOW-based TLS, the PPN embryos need not be removed, and the embryos, co-cultured with PPN embryos, may benefit through the ILIAP, ITI-H4, and keratins secreted from the PPN embryos. The G4 embryos (except PPN-derived) would do harm to the development of other embryos through KLKB1 and VTDB secretion.

Eight proteins were found to be commonly secreted by the PPN embryos and G4 embryos (**Supplementary Table 2**); however, we could not find any evidence to support the potential detrimental function of these eight proteins. This might further support our conclusion that PPN embryos could be beneficial for improving the development of the neighboring embryos, likely through the anti-inflammatory effect of ILIAP, ITI-H4, and keratins (**Figure 3**), without exerting any negative effects.

The present study has some limitations. First, our research subjects comprised of only Chinese patients with group-cultured embryos in TLS. Therefore, the universality and extrapolation of our findings to other cohorts may not be possible. Second, the microenvironment in the TLS is very complicated; apart from proteins and metabolites, the embryos can also secrete extracellular vehicles (EVs) to the group culture microenvironment (23). EVs have been proved to contain proteins and miRNAs that play an important role in embryo implantation. In the current study, we only analyzed the secretory proteins in the culture medium, without taking into account the potentially huge influence of the EV miRNAs on the neighboring embryos (24). However it is so hard to extract enough exosomes from the limited culture medium in the TLS. Therefore, further studies are warranted to determine the effect of the EVs derived from the G4 embryos on the development of neighboring embryos.

Nonetheless, our study also has some strengths. This is the first study to clarify the inclusion principles of human embryos in the WOW-based TLS system, which can guide clinical procedure. Second, former studies on embryo group culture mainly used bovine or mouse embryos, and lacked observations on human embryos. Our study overcomes this gap and confirms the importance of autocrine/paracrine factors in human embryo development.

## Conclusion

The present research proposes the inclusion principles of human embryos in the WOW-based TLS system: (1) the PPN embryos need not be removed; rather, the embryos co-cultured with the PPN embryos may benefit through the ILIAP, ITI-H4, and keratins secreted from the PPN embryos. (2) The number of G4 embryos should be no more than (≤) one to avoid their negative effects (potentially mediated by the secreted KLKB1 and VTDB proteins) on the development of other embryos.

## Data Availability Statement

The original contributions presented in the study are included in the article/**Supplementary Material**. Further inquiries can be directed to the corresponding author.

## Ethics Statement

The studies involving human participants were reviewed and approved by Ethical Review Committee of Peking University first hospital. The patients/participants provided their written informed consent to participate in this study. Written informed consent was obtained from the individual(s) for the publication of any potentially identifiable images or data included in this article.

## Author Contributions

YW and YX conceived the study and designed the major experiments. YW and SW analyzed data. XQ and YK contributed to materials and methods. YW and YX wrote the manuscript. All authors contributed to the article and approved the submitted version.

## Funding

This study is supported by the following grants: Beijing Municipal Natural Science Foundation (7194324) and National Natural Science Foundation of China (81901475).

## Conflict of Interest

The authors declare that the research was conducted in the absence of any commercial or financial relationships that could be construed as a potential conflict of interest.

## Publisher’s Note

All claims expressed in this article are solely those of the authors and do not necessarily represent those of their affiliated organizations, or those of the publisher, the editors and the reviewers. Any product that may be evaluated in this article, or claim that may be made by its manufacturer, is not guaranteed or endorsed by the publisher.
