# Supplementary material for: The Inclusion Principles of Human Embryos in the WOW-Based Time-Lapse System: A Retrospective Cohort Study
Source: Front Endocrinol (Lausanne). 2021 Jul 26;12:549216. doi: 10.3389/fendo.2021.549216 (PMC8350438; doi:10.3389/fendo.2021.549216)
Supplement: Supplementary file 1 [file Table_1.docx]

**Table S1: the sub-group analysis**

| No. of embryos | N | Blastocyst formation rate | Transplantable blastocyst formation rate | High quality blastocyst formation rate |
| --- | --- | --- | --- | --- |
| 8≤No.＜12 | | | | |
| A | 30 | Ref | Ref | Ref |
| B | 20 | 0.36 (0.13, 0.59) 0.0034 | 0.28 (0.05, 0.51) 0.0177 | 0.27 (0.09, 0.45) 0.0048 |
| C1 | 18 | 0.11 (-0.20, 0.42) 0.1373 | 0.09 (-0.18, 0.36) 0.3138 | -0.02 (-0.21, 0.16) 0.8149 |
| C2 | 6 | -0.15 (-0.51, 0.21) 0.4150 | -0.10 (-0.45, 0.25) 0.5817 | -0.13 (-0.41, 0.14) 0.3505 |
| 12≤No.＜16 | | | | |
| A | 61 | Ref | Ref | Ref |
| B | 25 | 0.22 (0.09, 0.35) 0.0013 | 0.30 (0.18, 0.42) <0.0001 | 0.24 (0.14, 0.33) <0.0001 |
| C1 | 22 | -0.05 (-0.19, 0.08) 0.4368 | -0.03 (-0.16, 0.10) 0.6378 | -0.05 (-0.15, 0.05) 0.3330 |
| C2 | 15 | -0.16 (-0.32, -0.01) 0.0441 | -0.20 (-0.35, -0.06) 0.0068 | -0.06 (-0.18, 0.06) 0.3086 |
| 16≤No.＜20 | | | | |
| A | 40 | Ref | Ref | Ref |
| B | 32 | 0.07 (-0.05, 0.19) 0.2317 | 0.09 (-0.02, 0.20) 0.1237 | 0.08 (-0.03, 0.19) 0.1515 |
| C1 | 21 | -0.03 (-0.16, 0.11) 0.7094 | -0.00 (-0.13, 0.12) 0.9709 | -0.01 (-0.13, 0.11) 0.8795 |
| C2 | 13 | -0.15 (-0.31, 0.01) 0.0471 | -0.15 (-0.30, 0.00) 0.0367 | -0.12 (-0.27, 0.02) 0.0486 |

The data in the table: effect size (95% CI) p value

adjusted factors：Female’s age (y); Basal Serum AMH(mIU/ml); BMI(kg/cm3);

Group A: control group, means that there are no Grade IV embryos; Group C1 means that there exist less than 1 Grade IV embryos(except Polypronuclear embryo); Group C2 means that there exist more than 1 Grade IV embryos(except Polypronuclear embryo) in the WOW based TLS system
