# Supplementary material for: The Inclusion Principles of Human Embryos in the WOW-Based Time-Lapse System: A Retrospective Cohort Study
Source: Front Endocrinol (Lausanne). 2021 Jul 26;12:549216. doi: 10.3389/fendo.2021.549216 (PMC8350438; doi:10.3389/fendo.2021.549216)
Supplement: Supplementary file 2 [file Table_2.docx]

**Table S2: the Uniprto ID , name and function of the protein secreted by the embryos with different grades.**

| **Grade** | **Uniprto** | **Gene** | **Protein** | **Function** |
| --- | --- | --- | --- | --- |
| Common shared | Q14624 | ITIH4 | Inter-alpha-trypsin inhibitor heavy chain H4 | ITI-H4 might play positive roles in the maintenance of  Th1/Th2 balance, contribute to anti-inflammation[1]. |
|  | P01042 | KNG1 | Kininogen-1 | HMW-kininogen plays an important role in blood coagulation by helping to position optimally prekallikrein and factor XI next to factor XII |
|  | P05543 | THBG | Thyroxine-binding globulin | Major thyroid hormone transport protein in serum |
|  | P14151 | LYAM1 | L-selectin | mediates cell adhesion by binding to glycoproteins on neighboring cells .Mediates the adherence of lymphocytes to endothelial cells of high endothelial venules in peripheral lymph nodes. |
|  | P02654 | APOC1 | Apolipoprotein C-I | Inhibitor of lipoprotein binding to the low density lipoprotein (LDL) receptor, LDL receptor-related protein, and very low density lipoprotein (VLDL) receptor. Associates with high density lipoproteins (HDL) and the triacylglycerol-rich lipoproteins in the plasma and makes up about 10% of the protein of the VLDL and 2% of that of HDL. Appears to interfere directly with fatty acid uptake and is also the major plasma inhibitor of cholesteryl ester transfer protein (CETP). Binds free fatty acids and reduces their intracellular esterification. Modulates the interaction of APOE with beta-migrating VLDL and inhibits binding of beta-VLDL to the LDL receptor-related protein. |
|  | P05155 | IC1 | Plasma protease C1 inhibitor | May play a potentially crucial role in regulating important physiological pathways including complement activation, blood coagulation, fibrinolysis and the generation of kinins. Very efficient inhibitor of FXIIa. Inhibits chymotrypsin and kallikrein |
| G1~G3 embryos | P35527 | K1C9 | Keratin, type I cytoskeletal 9 | May serve an important special function either in the mature palmar and plantar skin tissue or in the morphogenetic program of the formation of these tissues. Plays a role in keratin filament assembly. |
|  | Q5XKE5 | K2C79 | Keratin, type II cytoskeletal 79 | expression increased in the epithelial cells of the uterine cavity, facilitating the embryo implantation. |
|  | Q9GZR7 | DDX24 | ATP-dependent RNA helicase | ATP-dependent RNA helicase. |
|  | P08571 | CD14 | Monocyte differentiation antigen CD14 | binds to monomeric lipopolysaccharide and delivers it to the LY96/TLR4 complex, thereby mediating the innate immune response to bacterial lipopolysaccharide (LPS) |
|  | P01834 | IGKC | Immunoglobulin kappa constant | membrane-bound or secreted glycoproteins produced by B lymphocytes. In the recognition phase of humoral immunity, the membrane-bound immunoglobulins serve as receptors which, upon binding of a specific antigen, trigger the clonal expansion and differentiation of B lymphocytes into immunoglobulins-secreting plasma cells |
|  | Q15849 | UT2 | Urea transporter 2 | Specialized low-affinity vasopressin-regulated urea transporter. Mediates rapid transepithelial urea transport across the inner medullary collecting duct and plays a major role in the urinary concentrating mechanism.  Involved in urea transport in rainbow trout embryo[2] |
|  | P02647 | APOA1 | Apolipoprotein A-I | Participates in the reverse transport of cholesterol from tissues to the liver for excretion by promoting cholesterol efflux from tissues and by acting as a cofactor for the lecithin cholesterol acyltransferase (LCAT). As part of the SPAP complex, activates spermatozoa motility. |
|  | P54108 | CRIS3 | Cysteine-rich secretory protein 3 | Neutrophil degranulation |
| G4 embryos | P02774 | VTDB | Vitamin D-binding protein | Involved in vitamin D transport and storage, scavenging of extracellular G-actin, enhancement of the chemotactic activity of C5 alpha for neutrophils in inflammation and macrophage activation.  negative correlations with fertility ranking in bull[3] |
|  | P03952 | KLKB1 | Plasma kallikrein | It releases bradykinin from HMW kininogen and may also play a role in the renin-angiotensin system by converting prorenin into renin.  Promotes the transform of ITI-H4 from the longer one to the shorter form ITI-H4 (ΔN688) which is a crucial inflammatory factor[1] |
|  | Q0VDD8 | DYH14 | Dynein heavy chain 14, | Dynein has ATPase activity; the force-producing power stroke is thought to occur on release of ADP. Involved in sperm motility; implicated in sperm flagellar assembly |
|  | P21754 | ZP3 | Zona pellucida sperm-binding protein 3 | Component of the zona pellucida, an extracellular matrix surrounding oocytes which mediates sperm binding, induction of the acrosome reaction and prevents post-fertilization polyspermy. |
|  | Q66K66 | TM198 | Transmembrane protein 198 | Promotes LRP6 phosphorylation by casein kinases and thereby plays a role in Wnt signaling. May be a membrane scaffold protein involved in the self-aggregation of LRP6 to further enhance its activity |
|  | Q5T013 | HYI | Putative hydroxypyruvate isomerase | Catalyzes the reversible isomerization between hydroxypyruvate and 2-hydroxy-3-oxopropanoate (also termed tartronate semialdehyde. |
|  | Q9H2T7 | RBP17 | Ran-binding protein 17 | May function as a nuclear transport receptor. |
|  | O15195 | VILL | Villin-like protein | Possible tumor suppressor. |
|  | P52746 | ZN142 | Zinc finger protein 142 | May be involved in transcriptional regulation |
|  | O43432 | IF4G3 | Eukaryotic translation initiation factor 4 | component of the protein complex eIF4F, which is involved in the recognition of the mRNA cap, ATP-dependent unwinding of 5'-terminal secondary structure and recruitment of mRNA to the ribosome. |
|  | Q96M34 | TEX55 | Testis-specific expressed protein 55 | No reference |
|  | P00739 | HPTR | Haptoglobin-related protein | Primate-specific plasma protein associated with apolipoprotein L-I (apoL-I)-containing high-density lipoprotein (HDL). Which mediates human innate immune Binds hemoglobin with high affinity and may contribute to the clearance of cell-free hemoglobin to allow hepatic recycling of heme iron[4]. |
|  | P07359 | GP1BA | Platelet glycoprotein Ib alpha chain | GP-Ib, a surface membrane protein of platelets, participates in the formation of platelet plugs by binding to the A1 domain of vWF, which is already bound to the subendothelium. |
|  | P01009 | A1AT | Alpha-1-antitrypsin | Irreversibly inhibits trypsin, chymotrypsin and plasminogen activator and inhibits insulin-induced NO synthesis in platelets, decreases coagulation time and has proteolytic activity against insulin and plasmin. |
|  | P02655 | APOC2 | Apolipoprotein C-II | Component of chylomicrons, very low-density lipoproteins (VLDL), low-density lipoproteins (LDL), and high-density lipoproteins (HDL) in plasma. Plays an important role in lipoprotein metabolism as an activator of lipoprotein lipase. |
|  | P02656 | APOC3 | Apolipoprotein C-III | Component of triglyceride-rich very low density lipoproteins (VLDL) and high density lipoproteins (HDL) in plasma; extracellularly, attenuates hydrolysis and clearance of triglyceride-rich lipoproteins (TRLs) |
|  | Q9UGM5 | FETUB | Fetuin-B | Required to prevent premature zona pellucida hardening before fertilization, probably by inhibiting the protease activity of ASTL, a protease that mediates the cleavage of ZP2 and triggers zona pellucida hardening |
|  | P12955 | PEPD | Xaa-Pro dipeptidase | Splits dipeptides with a prolyl or hydroxyprolyl residue in the C-terminal position. Plays an important role in collagen metabolism |
|  | P04278 | SHBG | Sex hormone-binding globulin | androgen transport protein. Specific for 5-alpha-dihydrotestosterone, testosterone, and 17-beta-estradiol. Regulates the plasma metabolic clearance rate of steroid hormones by controlling their plasma concentration. |
| PPN embryos | P15151 | PVR | Poliovirus receptor | NK cell adhesion and triggers NK cell effector functions;  Involved in development of the central nervous system[5]:  Loss of Nectin-2 Correlates with Severe Spermatozoan Head and Midpiece Malformation, mpaired Binding to the Zona Pellucida, and Oocyte Penetration[6] |
|  | P35908 | K22E | Keratin  type II cytoskeletal 2 epidermal | keratinocyte activation, proliferation and keratinization |
|  | Q14532 | K1H2 | Keratin, type I cuticular Ha2 | keratinocyte activation, proliferation and keratinization |
|  | P01008 | ANT3 | Antithrombin-III | Most important serine protease inhibitor in plasma that regulates the blood coagulation cascade. |
|  | Q9NPH3 | IL1AP | Interleukin-1 receptor accessory protein | Coreceptor for IL1RL2 in the IL-36 signaling system. Coreceptor with IL1R1 in the IL-1 signaling system. Associates with IL1R1 bound to IL1B to form the high affinity interleukin-1 receptor complex which mediates interleukin-1-dependent activation of NF-kappa-B and other pathways.  Associates with secreted ligand-bound IL1R2 and increases the affinity of secreted IL1R2 for IL1B; this complex formation may be the dominant mechanism for neutralization of IL1B by secreted/soluble receptors Enhances the ability of secreted IL1R1 to inhibit IL-33 signaling[7, 8] |

1. L, L., et al., *Opposing roles of inter-α-trypsin inhibitor heavy chain 4 in recurrent pregnancy loss.* EBioMedicine, 2018. **37**: p. 535-546.

2. CM, P. and W. PA, *The mechanisms of urea transport by early life stages of rainbow trout (Oncorhynchus mykiss).* The Journal of experimental biology, 2000. **203**: p. 3199-207.

3. AGA, V., et al., *Proteomic landscape of seminal plasma associated with dairy bull fertility.* Scientific reports, 2018. **8**(1): p. 16323.

4. Nielsen, M.J., et al., *Haptoglobin-related protein is a high-affinity hemoglobin-binding plasma protein.* Blood, 2006. **108**(8): p. 2846-9.

5. JK, D., et al., *Poliovirus replication and spread in primary neuron cultures.* Virology, 2005. **340**(1): p. 10-20.

6. S, M., et al., *Loss of nectin-2 at Sertoli-spermatid junctions leads to male infertility and correlates with severe spermatozoan head and midpiece malformation, impaired binding to the zona pellucida, and oocyte penetration.* Biology of reproduction, 2003. **69**(4): p. 1330-40.

7. Smith, D.E., et al., *The soluble form of IL-1 receptor accessory protein enhances the ability of soluble type II IL-1 receptor to inhibit IL-1 action.* Immunity, 2003. **18**(1): p. 87-96.

8. Jensen, L.E., et al., *IL-1 signaling cascade in liver cells and the involvement of a soluble form of the IL-1 receptor accessory protein.* J Immunol, 2000. **164**(10): p. 5277-86.
